# 

**Published:** 1894-12-29

**Authors:** 


					p HOSPITAL. y  usax ggy a^,.
^ PRICE TWOPENCE uTT^ 1W IS5^ W,TH EXTRA nursing supplement.
Mi, vol. XVII. Dec. 29th, 1894. HT fH JL
Ditto per Annum, 8s. 6d. post free.
^ NORFOLK*** NORWICH HOSPl.TAJ.
No. 43lV Vol. XVII. WITH EXTRA NURSING SUPPLEMENT. Satubday, Dec. 29tii, 1894

				

